# Effect of Genetic Polymorphism of Bovine β-Casein Variants (A1 and A2) on Yoghurt Characteristics

**DOI:** 10.3390/foods13244135

**Published:** 2024-12-20

**Authors:** Bibiana Juan Godoy, Idoia Codina-Torrella, Antonio-José Trujillo Mesa

**Affiliations:** 1 Centre d’Innovació, Recerca i Transferència en Tecnologia dels Aliments (CIRTTA), TECNIO (CERTA-UAB), Departament de Ciència Animal i dels Aliments, Facultat de Veterinària, Universitat Autònoma de Barcelona (Cerdanyola del Vallès), 08193 Barcelona, Spain; toni.trujillo@uab.cat; 2 Departament d’Enginyeria Agroalimentària i Biotecnologia, Universitat Politècnica de Catalunya, Edifici D4C, Esteve Terradas, 8, Castelldefels, 08860 Barcelona, Spain; idoia.codina@upc.edu

**Keywords:** A2 β-casein, genetic polymorphism, yoghurt, acid coagulation, physicochemical, sensory characteristics

## Abstract

The present study aims to evaluate the physicochemical and sensory characteristics of A2 yoghurts (made with A2A2 β-CN milk), in comparison with Control yoghurts (elaborated from conventional milk, a mixture of A1 and A2 β-CN milk). The pH, acidity, water-holding capacity, spontaneous syneresis, firmness and color of yoghurts were monitored during their cold storage (4 °C) for 35 days. Two independent sensory tests (with expert judges and consumers) were also performed. The A2 yoghurts showed only minor differences in some of their physicochemical and sensory characteristics compared to those made with conventional milk. At specific storage times, the A2 yoghurt exhibited higher levels of acidity, luminosity (L*) and firmness, compared to the Control. No differences were observed in the growth curves of the starter (*Lactobacillus delbrueckii* subsp. *bulgaricus* and *Streptococcus salivarius* subsp. *thermophilus*) during the yoghurt production, nor in the water-holding capacity or spontaneous syneresis of the two types of gels. Regarding the sensory evaluation of samples, the A2 yoghurts were described as firmer and more adherent (by the expert panel), and brighter and more homogeneous (by the consumers) than the Control. In all cases, both consumers and expert sensory panels showed a preference for the A2 yoghurts. Therefore, these results demonstrate the suitability of A2A2 β-CN milk for producing yoghurts with similar characteristics to those obtained with conventional milk.

## 1. Introduction

Milk proteins, particularly caseins, play a fundamental role in acid gelation processes. Bovine milk typically contains about 3–4% protein, with four types of caseins (CNs) (α_s1_-CN, α_s2_-CN, β-CN and κ-CN) accounting for ~80% of the total protein. The remaining 20% corresponds to whey proteins (WPs), which mainly consist of β-lactoglobulin (β-Lg) and α-lactalbumin (α-La) [1]. The β-CN constitutes about 40% of the CNs and has different genetic variants, of which A1 and A2 represent the most common [2]. The main difference between the A2 (the ancestral variant) and A1 variants concerns amino acid position 67 of the β-CN, which corresponds to proline or histidine, respectively [3]. These differences involve the alteration of the technofunctionality of milk (gelation, water binding, etc.) and may also have implications for human digestive processes [4,5,6]. Some studies have indicated that β-CN A1 digestion releases a greater quantity of beta-casomorphine-7 (BCM-7) than β-CN A2 [7,8] and EFSA has declared a cause–effect relationship between the intake of BCM-7 and gastrointestinal tract disturbances [9]. Based on these findings, A2A2 β-CN milk continues to gain prominence in the global market, and research on milk protein polymorphisms is on the rise [4].

Yoghurt is one of the most popular fermented dairy products, which is produced by the fermentation of milk by *Lactobacillus delbrueckii* subsp. *bulgaricus* and *Streptococcus thermophilus*. The physicochemical and sensory characteristics of yoghurts (i.e., the firmness, the expelled whey or the color) are crucial for consumer acceptance [10]. Milk used for yoghurt production is typically heat-treated at temperatures of 90–95 °C for 5–10 min and then cooled to ~43 °C, before the addition of the starter culture. The gel is formed by the activity of the starter bacteria, which mainly produce lactic acid from lactose and consequently decrease the pH of the milk until the isoelectric point of the caseins (pH ~ 4.6) [10]. The reduction in electrostatic repulsion between the charged micelles causes casein–casein attractions and results in the gelation of the matrix [11]. Additionally, the heating of milk also results in a β-Lg denaturalization and subsequent attachment to the surface of CN micelles. Although different processing parameters are decisive in the final characteristics of these gels [12,13], the genetic polymorphism of milk proteins has also been reported to affect the gelation properties of milk [4]. Daniloski et al. [14] demonstrated that acid gels obtained from skimmed milk containing A2A2 β-CN showed a lower water-holding capacity and elastic modulus, and also presented a softer microstructure when compared to A1A1 and A1A2 milk gels. These authors also reported that the gelation times of A1A1 and A1A2 milk were shorter than those obtained with A2A2 β-CN milk. Nguyen et al. [6] also described longer coagulation times in acid gels produced with A2A2 β-CN milk and reported that those gels exhibited a less dense protein network (gels were more porous and contained shorter protein strains), in comparison to the yoghurts produced with A1 β-CN milk. Juan and Trujillo [15] found that acid gels obtained with milk containing only β-CN A2A2 were denser and showed higher latency times compared to those elaborated with conventional milk (tank milk of cows with β-CN A2A1, A1A1 and A2A2 genotypes). These authors also described a tendency towards lower water-holding capacity in A2 gels, although the differences between the two types of tank milks (A2A2 and conventional) were not statistically significant. All these findings suggest that the gelation properties of milk may be related to the primary amino acid structure of its proteins. In the absence of sufficient knowledge of the effect of β-CN polymorphism on the physicochemical and sensory characteristics of yoghurts, this study aimed to evaluate the impact of using tank milk with the A2A2 β-CN genotype on the physicochemical and sensory characteristics of yoghurts (stored at 4 °C for 35 days), in comparison to yoghurts made from conventional milk (a mixture of tank milk with A1A1 and A1A2 β-CN).

## 2. Materials and Methods

### 2.1. Milk Collection

In the current study, two types of tank milk were used for yoghurt production (milk from cows with A2A2 β-CN genotype and milk from cows with A1A2 and A1A1 β-CN genotypes, as Control). The milks were obtained from January to May 2022 from a Catalan farm (Manlleu, Barcelona, Spain), on three different occasions. The cows used in the present study were selected according to their casein genotypes and days in milk and number of lactation, trying to make them as similar as possible in both groups. All the populations were of the Holstein–Friesian breed. The genetic information of the individual cows was obtained from CONAFE (Confederation of Spanish Friesian Associations, Spain) (Table 1).

### 2.2. Yoghurt Production

The tank milks were received in the Food Technology Plant Service of the Universitat Autònoma de Barcelona (SPTA, UAB, Bellaterra, Barcelona, Spain). After standardization at ~3.5% of fat, the milks were homogenized at 20 MPa and 65 °C with Homolab 2.20 equipment (FBF, Parma, Italy) and pasteurized at 90 °C for 5 min in a tubular heat-exchanger (ATI, Granollers, Barcelona, Spain). Then, the milks were cooled to ~43 ± 2 °C and inoculated with 3% of the starter culture (*Lactobacillus delbrueckii* subsp. *bulgaricus* and *Streptococcus salivarius* subsp. *thermophilus*, Yo-Mix 300 LYO 10 DCU, Danisco France SAA, Paris, France). Then, the containers were incubated at this temperature for ~4 h, until the samples reached a pH ~ 4.6. After the fermentation, the yoghurts were stored at ~4 °C, in a chamber, until their evaluation. Their physicochemical and sensory analyses were performed at 1, 7, 14, 21, 28 and 35 days of storage. Three independent productions of yoghurts were made.

### 2.3. Physicochemical Parameters

#### 2.3.1. Total Solids, pH, Protein Content and Acidity

The total solids were analyzed in triplicate (day 1 of storage) following the standard method for the determination of the total solids content of milk, proposed by ISO 6731:2010/IDF 21:2010 [16]. The results were expressed as grams of total solids per 100 g of sample. The protein content of the yoghurts was determined by the Kjeldahl method, according to ISO 8968-1:2014/IDF 20-1: 2014 [17]. The conversion factor of nitrogen to protein corresponded to 6.38. The measures were performed in triplicate and the results were expressed as grams of protein per 100 g of sample. The pH value and the acidity of the yoghurts were determined over their storage (at days 1, 7, 14, 21, 28 and 35). The pH value was measured in duplicate using a Crison basic 20 pHmeter (Crison Instruments, Barcelona, Spain). Titratable acidity was determined in duplicate according to the method reported by Akalın et al. [18] and expressed as milligrams of lactic acid per 100 g of yoghurt.

#### 2.3.2. Water-Holding Capacity (WHC) and Spontaneous Syneresis

For determining the WHC of the yoghurts, ~40 mL of milk were placed in different centrifuge tubs and then inoculated with the starter culture (3%). The tubes were kept at 43 °C until they reached pH ~ 4.6. Then, the tubes were placed in a cold chamber (at 4 °C) and sampled after 1, 7, 14, 21, 28 or 35 days. For each day of sampling, three different tubes of each type of sample were warmed to 20 °C and then centrifuged (Sigma 4K15, Sigma Osterode am Harz, Germany) at 5000× *g* for 20 min at 20 °C. Afterward, the whey of each tube was accurately removed from the gel and weighed. The WHC was calculated as the grams of expelled whey per 100 g of the initial sample.

Spontaneous syneresis was calculated through the extraction of the whey that was spontaneously separated from the gel during the storage time of the yoghurts (1, 7, 14, 21, 28 and 35 days) [19]. The whey of each container was accurately removed by decanting. The spontaneous syneresis was expressed as grams of decanted whey per 100 g of yoghurt. The analyses were performed with three independent units of each type of sample.

#### 2.3.3. Color and Texture

Color analyses of the yoghurts were conducted with a Hunter Lab colorimeter (MiniScan XETM, Hunter Associates Laboratory Inc., Reston, VA, USA). The color coordinates were measured with a standard observer angle of 10°, with a reference D65 illuminant. The colorimeter was previously calibrated with standard black and white tiles. Data were acquired in the CIElab color space, through the three tristimulus coordinates: L* (luminosity), a* (red–green) and b* (blue–yellow). The total color differences (AE*) were calculated according to the following equation [20]:

ΔE* = (ΔL*^2^ + Δa*^2^ + Δb*^2^)^0.5^ (1)

Textural analyses of the yoghurts were conducted using a TA-XT2 Texture Analyzer (Texture Analyser, Stable Micro Systems, Godalming, UK), equipped with a cylindrical probe of 10 mm in diameter. The firmness (maximum force in compression) of the samples was determined through a penetration test, by applying a constant velocity of 1 mm/s to a depth of 10 mm. The samples were previously prepared in different containers of 55 mm in diameter, which were stored at 4 °C after being analyzed. Force–distance curves were obtained by using the software Exponent 6.0.6.0 (Texture Technologies Corp., Hamilton, MA, USA). The color and texture analyses were performed with three independent units of each type of sample.

### 2.4. Microbiological Analyses

Selective enumeration of the starter culture was also performed. *S. thermophilus* and *L. bulgaricus* were counted in the selective media M17 and MRS agar, under anaerobic conditions, at 37 °C for 24 and 48–72 h, respectively. Molds and yeasts were enumerated in Rose Bengal Agar and incubated at 30 °C for 48 h. All media were purchased from Oxoid Ltd. (Basingstoke-Hampshire, UK). Microbiological analyses of the samples were performed in duplicate every 7 days, during the 35 days of storage. The results were expressed as a log of colony-forming units (CFUs) per gram of yoghurt.

### 2.5. Sensory Characteristics

Fifteen trained assessors from the staff of the Universitat Autònoma de Barcelona were preselected based on their previous experience of the evaluation of yoghurts. At 15 days of their cold storage, samples (Control and A2) were presented to the panelists at the same time. The assessors were asked to identify sensorial differences (syneresis, color, brightness, firmness, adherence, gel homogeneity, creaminess, viscosity, odor, acidity, flavor intensity and off flavor) of the A2 samples in comparison to the Control on a 7 point scale, with −3 and 3 points corresponding to very considerable differences, −2 and 2 to considerable differences, −1 or 1 points to minimal differences and 0 points to unnoticeable differences. The algebraic sign, i.e., negative or positive, indicates a lower or greater perception of the panelists. Finally, the expert assessors were asked to indicate their preference between the samples.

A consumer test was also performed, with a panel of 50 consumers (respondents were 37 women and 13 men, from ~20 to ~60 years). They were asked to evaluate the yoghurts considering the same attributes included in the sensory test conducted by the expert panel and to evaluate each sample on a scale of 7 points (from 1 to 7), where 1 corresponded to nothing and 7 to extremely. The consumers were also asked to evaluate their preference of the yoghurts according to a hedonic scale of 9 points (from 1 to 9), where 1 corresponded to I really dislike it and 9 corresponded to I really like it.

### 2.6. Statistical Analyses

The data on the yoghurts were compared by Student’s *t*-test for independent samples on each storage day. Differences during the storage period in every type of yoghurt were evaluated by an analysis of variance (ANOVA, IBM SPSS Statistics 25, Armonk, New York, NY, USA) and the significant differences between means were assessed by Tukey’s test, at a significance level of *p* ≤ 0.05. The whole experiment was repeated three times independently.

## 3. Results and Discussion

### 3.1. Genetics of Cows Included in This Study

The milk used in the present study was selected according to the genotypes of cows, days in milk and number of lactation, trying to make the samples as similar as possible in both groups (Table 1), since among other factors, the coagulation properties of milk are greatly influenced by the genotypes of β-CN, κ-CN and β-LG [12,21,22,23]. In the current study, the Control milk corresponded to a mixture of tank milk from animals with A1A2 (in a major proportion) and A1A1 genotypes of β-CN, to imitate as much as possible the genotypic reality of current conventional Friesian breed milk [15]. The milk used for the production of A2 yoghurts (A2 milk) was only constituted by the A2A2 variant of the β-CN. As regards κ-CN, only three genetic variants were found in the A2 milk (AB > AA ~ BB), whereas the Control showed a major versatility, which corresponded to AA = BE > AE > AB > BB > EE. In fact, Comin et al. (2008) mentioned that the E allele rarely appeared in milk in conjunction with the β-CN A2A2. This allele was related to bad rennet coagulation properties [24]. Concerning the β-LG, three types of variants were found in both milks (Control and A2), of which the AB allele corresponded to the most predominant. The AA and BB alleles exhibited the following tendency: BB > AA and AA > BB for the Control and the A2 milks, respectively. As seen, the A variant of β-LG is more frequent than the B variant in Holstein–Friesian cows, which is associated with better gelation properties [22].

### 3.2. Total Solids and Protein Content

No significant differences (*p* > 0.05) were observed in total solids and protein content between the Control and A2 yoghurts. The respective values were 11.85 ± 0.34 and 12.16 ± 0.19 g of total solids/100 g of sample and 3.14 ± 0.19 and 3.24 ± 0.05 g of protein/100 g of sample. These findings were consistent with previous studies, which reported no significant differences in total solids and protein content when comparing bovine milk with these two variant types of the β-CN [6,15,25].

### 3.3. Microbiological Counts of Starters

**Figure 1:**
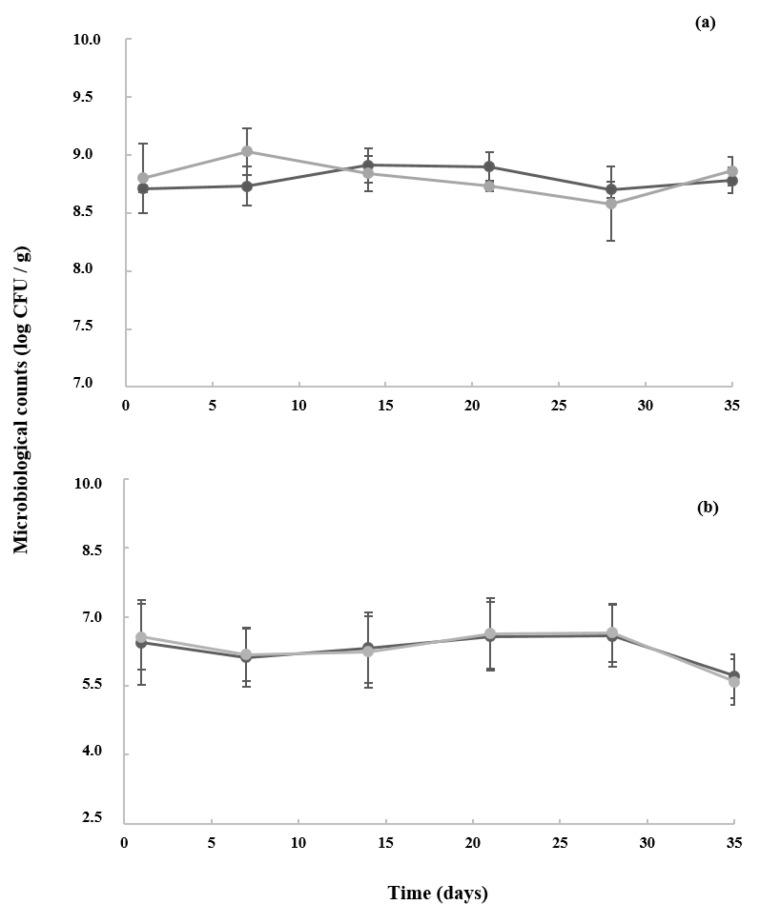
Microbiological counts of starters (**a**) *S. thermophilus* and (**b**) *L. bulgaricus* of Control and A2 yoghurts during their storage. Control (●): Control, yoghurt made with milk from cows with A1A2 and A1A1 β-CN genotypes; A2 (●): yoghurt made with milk from cows with A2A2 β-CN genotype.

Figure 1 shows the evolution of the total counts of *S. thermophilus* and *L. bulgaricus* during the storage of the yoghurts. As expected, molds and yeasts were not detected in any sample.

These findings suggested that the evolution of *S. thermophilus* and *L. bulgaricus* counts in both types of yoghurts (Control and A2) was not influenced (*p* > 0.05) by the type of milk. At day 1, the total counts of the starters corresponded to 8–9 and 5–6 log CFU/mL for *S. thermophilus* and *L. bulgaricus*, respectively. The initially low counts of *L. bulgaricus* could be attributed to the insufficient protocooperation between these two bacteria at the beginning of fermentation. However, from day 7 onwards, the growth curve of these bacteria showed a positive tendency, which was attributed to the more optimal growing conditions of the medium (acid pH and low temperatures) [26,27]. *L. bulgaricus* is responsible for the post-acidification of yoghurts from half of their storage, which would justify the increase in total acidity in yoghurts after this point (see Table 2). During their storage, in both types of yoghurts (Control and A2), the microbiological counts of the starters were aligned to those described in the quality standard for yoghurt, which required doses ≥ 7 log CFU of the microorganisms constituting the starter culture by gram of product [28].

### 3.4. pH and Acidity

Table 2 shows the pH values and the acidity of both yoghurt types. During the gel formation, the production of several organic acids leads to a decrease in the pH value, resulting in a protein denaturation and the coagulation of milk [29]. As observed, in general, the pH of all the yoghurts (Control and A2) decreased over the storage, while the titrated acidity showed an opposite tendency. At day 1, the pH value was similar for both types of yoghurts (Control and A2) and corresponded to 4.51 ± 0.02 and 4.57 ± 0.03, respectively. These values remained stable (*p* > 0.05) until 21 (Control) and 14 (A2) days of storage. From that point, the pH dropped in both types of yoghurts, probably due to the bacterial activity and subsequent acid production [26,30]. As shown in Table 2, at day 35, both products exhibited a similar pH value (*p* > 0.05).

Acidity is one of the most important quality parameters in yoghurts, which compromises and determines their specific sensory profile and shelf life [31]. In the current study, the titratable acidity increased over storage in both types of yoghurts, in accordance with the decrease in their pH value. In these samples (Control and A2 yoghurts), the acidity increased until 7 and 14 days of storage, respectively, and then, these values remained stable (Table 2). Significant higher values of acidity (*p* < 0.05) were observed in the A2 yoghurts from day 21 of storage onwards, compared with the Control. It is worth noting that Wang et al. [32] also reported significantly higher values of titratable acidity in A2 fermented milk gels, in comparison to those produced with A1 milk. These authors demonstrated that during the fermentation step, a larger amount of proteins were hydrolyzed in the A2A2 β-CN bovine milk, which led to the production of more small peptides, in comparison to the A1 milk. Considering these findings, differences in titratable acidity observed in the current study between the Control and A2 yoghurts might be attributed to the variations in the acidic by-products (peptides, amino acids, etc.) of these samples and also to the different interaction of these by-products with the milk constituents present in yoghurts during their storage. Although the pH of the samples was similar (Table 2), these differences would be only detected in the titratable acidity determination.

### 3.5. Water-Holding Capacity and Spontaneous Syneresis

Water-holding capacity (WHC) is an important indicator of yoghurt’s capacity to retain whey. WHC is crucial for yoghurt quality, as it directly influences overall consumer acceptance [18]. Higher values of WHC ensure that the yoghurt maintains its stability and firmness and prevents syneresis (the undesirable expulsion of whey), which can negatively impact the product’s appearance and texture [18]. As shown in Table 3, the WHC values for both the Control and A2 yoghurts were similar (*p* > 0.05) and remained stable throughout the storage (*p* > 0.05). These results demonstrated the initial stability of both types of gels (day 1) and also their ability to retain water for ~ 35 days. The spontaneous syneresis of both the Control and A2 gels (Table 3) showed no differences (*p* > 0.05) depending on the type of sample (Control or A2) or the storage time. These results suggested that the genetic variant of β-CN would not affect the WHC of these yoghurts and, subsequently, their spontaneous syneresis. In previous studies, Juan and Trujillo [15] reported that non-significant differences were observed in the water-holding capacity of acid gels obtained with A2A2 β-CN tank milk and conventional tank milk. Wang et al. [32] also did not observe differences in the WHC of several A1 and A2 fermented milks, and also described WHC percentages of ~84 to ~85%. A different outcome was reported by Daniloski et al. [14], who described that the WHC of yoghurts produced by A2A2 milk was significantly lower than yoghurts elaborated by A1A1 and A1A2 milk.

### 3.6. Color and Texture

**Figure 2:**
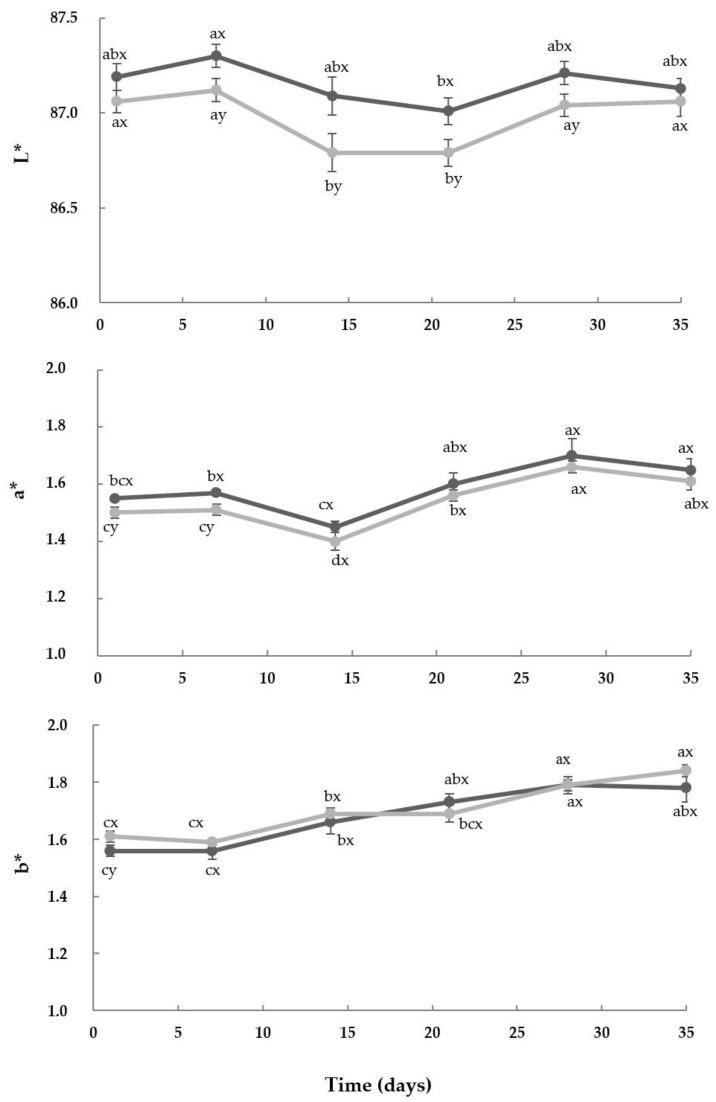
Evolution of color coordinates of yoghurt samples during their storage at 4 °C. Control (●): yoghurt made with milk from cows with A1A2 and A2A2 β-CN genotypes; A2 (●): yoghurt made with milk from cows with A2A2 β-CN genotype; L*: luminosity, a*: green–red, b*: blue–yellow. Data are means ± standard deviation. ^a,b,c^ For each color parameter, type of yoghurt and during the storage time, values with different superscripts differed significantly (*p* < 0.05). ^x,y^ For each day of storage and color parameter, values with different superscripts indicate significant differences (*p* < 0.05) between the Control and A2 samples.

Figure 2 summarizes the colorimetric analysis results of the yoghurts during their storage. As observed, the color parameters for each type of yoghurt (Control and A2) remained relatively constant over time, but showed significant differences according to the type of milk. In comparison to the Control, the A2 yoghurts exhibited the highest luminosity (L*) (*p* < 0.05) at 7, 14, 21 and 28 days of storage. These results indicated that these samples were brighter. The higher L* values in the yoghurts made from milk containing A2A2 β-casein might be related to the higher values of titratable acidity determined in the A2 yoghurts over the storage (which were significant (*p* < 0.05) from day 21), in comparison to their A1 homologs. Increased acidity can lead to changes in the microstructure of the yoghurt, potentially causing a more uniform distribution of particles and reducing light scattering [33]. In consequence, these samples exhibited higher L* values, being brighter or with a lighter appearance.

**Figure 3:**
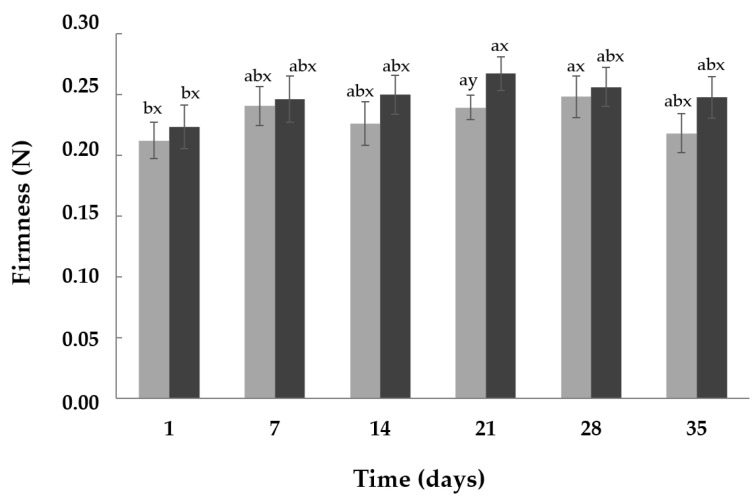
Firmness of yoghurts during their storage at 4 °C. Control (●): yoghurt made with milk from cows with A1A2 and A2A2 β-CN genotypes; A2 (●): yoghurt made with milk from cows with A2A2 β-CN genotype; N: Newtons; Data are means ± standard deviation. ^a,b^ For each type of yoghurt and during the storage time, values with different superscripts differed significantly (*p* < 0.05). ^x,y^ For each day of storage, values with different superscripts indicate significant differences (*p* < 0.05) between the Control and A2 samples.

The a* (green–red) and b* (blue–yellow) coordinates indicated positive values in both types of yoghurt throughout their storage and contributed to a color tendency toward red and yellow, respectively. During the first 7 days of storage, the A2 yoghurts showed higher a* values than the Control, but from this point, these samples did not exhibit significant differences (*p* > 0.05) (Figure 3). In contrast, on day 1, the b* coordinate presented the highest values (*p* < 0.05) in the Control. This parameter gradually increased in both yoghurt types over time and did not show statistical differences (*p* < 0.05) among them. Similar results were previously described in the literature, in which the A2 fermented gels presented higher brightness and redness, in comparison to their homologs produced with A1 milk [32]. If comparing each type of yoghurt (Control and A2) after production (day 1) with their corresponding homologs at day 35, their global color differences (ΔE*) corresponded to 0.25 ± 0.01 and 0.31 ± 0.08, respectively. These results evidenced that the overall color of the yoghurts slightly changed over their storage. In addition, these findings also revealed that these color differences would not be visible to the human eye, since the AE* was <3 in every case [34]. The calculated overall color differences (ΔE*) between the Control and A2 yoghurts corresponded to 0.17 ± 0.06 (day 1) and 0.18 ± 0.01 (day 35), respectively, which suggested that the color differences among the yoghurts produced with milk of different β-CN genotypes would not be noticed by consumers. In fact, these results were in line with those observed in the sensory evaluation of the samples, as described in Section 3.7.

Texture is one of the most important quality characteristics of yoghurts, with regard to the sensory perception of the product [10]. As observed in Figure 3, in this study, the genotype of β-CN slightly influenced the firmness of the yoghurts (Control and A2). In general, the firmness of both types of yoghurts showed similar values over the storage, although the A2 samples exhibited a tendency towards higher values for this attribute. Note that at day 21, the A2 yoghurts were significantly (*p* < 0.05) firmer than the Control. The firmness of yoghurts depends on the characteristics of the proteins involved in the gel network, including their profile and content, and the number of protein–protein bonds formed between the CNs, which are generated in protein re-arrangement during the yoghurt’s production [10,35].

In our case, no significant differences (*p* > 0.05) were observed between the protein content of the Control and the A2 milks. These results can indirectly prove the existence of a significant difference in the structure of the protein micelles of these two types of yoghurt. As reported by Wang et al. [32], the adoption of greater polyproline-II helix in A2 β- casein could influence the aggregation of proteins and consequently determine the gel structure, resulting in changes in the gelation properties of the fermented milk.

### 3.7. Sensory Evaluation of Yoghurts

**Figure 4:**
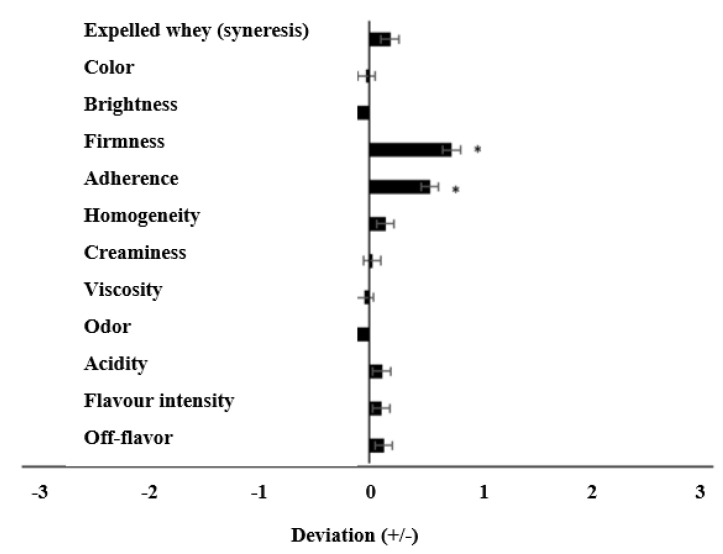
Scores obtained in the sensory evaluation of Control and A2 yoghurts, by the expert panel, at 15 days of storage. The Control sample was considered as the reference. Control: yoghurt made with milk from cows with A1A2 and A2A2 β-CN genotypes. A2: yoghurt made with milk from cows with A2A2 β-CN genotype.* *p* ≤ 0.05.

Figure 4 presents the results obtained in the sensory evaluation of the Control and A2 yoghurts, which were conducted by expert panelists. As observed, some attributes were described to be different in the A2 yoghurts, in comparison to the Control. However, in all cases, these differences were minor (with scores lower than ±1), indicating only slight differences among them. Only two attributes (firmness and adherence) of the A2 yoghurt showed a significant positive deviation (*p* < 0.05) compared to the Control, which was aligned with the findings obtained in the instrumental test (see Section 3.6). While 45.5% of the expert assessors expressed a preference for the A2 yoghurt, ~24.4% opted for the Control. This preference was mainly attributed to the texture of the yoghurts. The rest of the assessors (30.1%) did not show a clear preference, noting that their preference for both products (A2 and Control) was similar.

**Figure 5:**
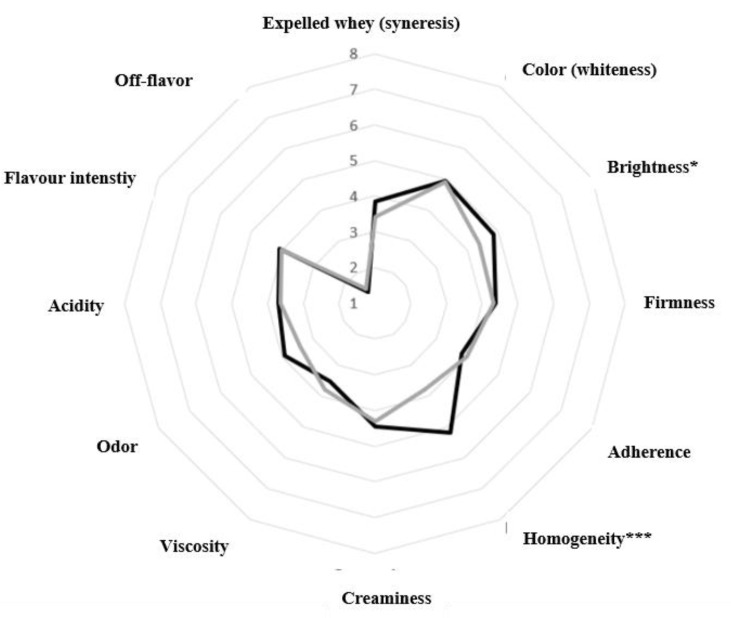
Scores obtained in the sensory evaluation of Control (●) and A2 (●) yoghurts, at 15 days of storage, conducted by the panel of consumers. Control: yoghurt made with milk from cows of A1A2 and A2A2 β-CN genotypes. A2: yoghurt made with milk from cows with A2A2 β-CN genotype. * *p* ≤ 0.05. *** *p* ≤ 0.001.

As shown in Figure 5, the consumers generally did not report significant differences (*p* > 0.05) between the two types of yoghurt (Control and A2) for most of the evaluated parameters. The consumers only differentiated the yoghurts by higher brightness and homogeneity scores in the A2 samples, consistent with the results of the instrumental tests. In the preference test, the A2 yoghurt received an average score of 7.16 ± 1.69 (I like it a lot), compared to 6.04 ± 1.62 for the Control (Slightly like it). This result indicated a modest consumer preference for the A2 yoghurts.

## 4. Conclusions

This study demonstrated that the β-CN genotype of milk slightly affected the quality characteristics (physicochemical and sensory) of yoghurts. Yoghurts produced with the β-CN A2 genetic variant were more acid, brighter and firmer, compared to the yoghurts produced with conventional milk (mixture of A1 and A2 variants), but these differences were observed only on specific days of storage. Interestingly, during the storage of the yoghurts, there were no differences in the growth curves of the bacterial starters, suggesting that casein polymorphism would not affect the growth of the main cultures of yoghurts (*S. bulgaricus* and *L. delbruekii*). Furthermore, the water-holding capacity and the spontaneous syneresis of these gels were unaffected by the type of milk. In the sensory evaluations, both the consumer and expert panels showed their preference for the A2 samples. Consumers found the A2 samples to be brighter and more homogeneous, and the expert panel noted that these yoghurts were firmer and more adherent than the Control. Consequently, these results indicated that the A2 yoghurts have similar characteristics to those produced with the A1 variant of β-CN and reinforce the potential production of A2 yoghurts in the dairy industry, by using conventional methods.

## Figures and Tables

**Table 1:** Information of the cows selected in the current study.

Milk ^1^	Lac ^3^	DIL ^4^	Number of Cows of Each Genetic Variant ^2^											
			β-CN			κ-CN						β-LG		
			A2A2	A1A2	A1A1	AA	BB	AB	BE	AE	EE	AB	BB	AA
C	2.1	118.8		12	3	4	1	2	4	3	1	8	6	1
A2	2.4	107.7	15			2	3	10				7	3	5

^1^ C: milk from cows with A1A2 and A1A1 β-CN genotypes; A2: milk from cows with A2A2 genotype. ^2^ β-CN: β-casein, κ-CN: κ-casein, β-LG: β-lactoglobulin; ^3^ Lac: Number of lactation ^4^ DIL: Days in lactation.

**Table 2:** pH value and titratable acidity (mg of lactic acid/100 g) of Control and A2 yoghurts during their storage at 4 °C.

	Yoghurt	Storage Time (Days) ^1^					
		1	7	14	21	28	35
pH	C	4.51 ± 0.02 ^a^	4.54 ± 0.05 ^a^	4.48 ± 0.02 ^ab^	4.50 ± 0.01 ^ab^	4.46 ± 0.03 ^b^	4.44 ± 0.04 ^b^
	A2	4.57 ± 0.03 ^a^	4.55 ± 0.03 ^ab^	4.48 ± 0.01 ^ab^	4.45 ± 0.03 ^b^	4.44 ± 0.03 ^b^	4.43 ± 0.04 ^b^
Acidity	C	83.83 ± 1.13 ^b^	89.65 ± 0.72 ^a^	91.41 ± 0.90 ^a^	90.35 ± 0.36 ^ay^	91.83 ± 0.34 ^ay^	91.16 ± 0.17 ^a,y^
	A2	84.55 ± 1.07 ^c^	90.58 ± 0.23 ^b^	92.58 ± 1.04 ^a^	94.62 ± 0.31 ^ax^	93.76 ± 0.82 ^ax^	94.79 ± 1.10 ^a,x^

^1^ Data are means ± standard deviation. C: yoghurt made with milk from cows with A1A2 and A2A2 β-CN genotypes; A2: yoghurt made with milk from cows with A2A2 β-CN genotype. ^a,b,c^ For each type of yoghurt and during the storage time, values with different superscripts differed significantly (*p* < 0.05). ^x,y^ For each day of storage, values with different superscripts indicate significant differences (*p* < 0.05) between the Control and A2 samples.

**Table 3:** Evolution of water-holding capacity (WHC, grams of expelled whey/100 g of yoghurt) and spontaneous syneresis (grams of whey/100 g of yoghurt) of Control and A2 yoghurts, during their storage at 4 °C.

	Yoghurt	Storage Time (Days) ^1^					
		1	7	14	21	28	35
WHC	C	65.47 ± 0.06 ^ax^	63.79 ± 0.05 ^ax^	61.83 ± 0.05 ^ax^	67.06 ± 0.05 ^ax^	65.01 ± 0.03 ^ax^	63.74 ± 0.04 ^ax^
	A2	63.31 ± 0.07 ^ax^	64.68 ± 0.04 ^ax^	59.46 ± 0.04 ^ax^	63.54 ± 0.03 ^ax^	64.61 ± 0.04 ^ax^	63.05 ± 0.02 ^ax^
Spontaneous syneresis	C	0.90 ± 0.14	1.04 ± 0.27	0.83 ± 0.24	0.85 ± 0.17	0.75 ± 0.14	0.62 ± 0.12
	A2	0.88 ± 0.14	0.64 ± 0.12	0.86 ± 0.11	1.09 ± 0.21	0.60 ± 0.09	0.75 ± 0.17

^1^ Data are means ± standard deviation. C: yoghurt made with milk from cows of A1A2 and A2A2 β-CN genotypes; A2: yoghurt made with milk from cows with A2A2 β-CN genotype. ^a^ For each type of yoghurt and during the storage time, values with different superscripts differed significantly (*p* < 0.05). ^x^ For each day of storage, values with different superscripts indicate significant differences (*p* < 0.05) between the Control and A2 samples.

## Data Availability

The original contributions presented in this study are included in the article. Further inquiries can be directed to the corresponding author.
